# Campbell Title Registrations to Date ‐ May 2019

**DOI:** 10.1002/cl2.1044

**Published:** 2019-08-06

Below is a list of all titles for systematic reviews or evidence and gap maps that have been accepted by the Editor of a Campbell Coordinating Group. When titles progress to protocol stage, the protocol is published in the Campbell Systematic Reviews journal.

The details of new titles are published in each issue of the journal and added to this page at the same time.

If you would like to receive a copy of the approved title registration form, please send an email to the Managing Editor of the relevant Coordinating Group.

## 1 BUSINESS AND MANAGEMENT

*Predictors of virtual team outcomes*, Iulia Cioca, Oana Fodor, Shannon Marlow, 13 February 2019.

*Multisource feedback and work performance*, Emilia Wietrak, Iulia Cioca and Jonny Gifford, 13 February 2019.

## 2 CRIME AND JUSTICE

*Police programs that seek to increase community connectedness for reducing violent extremism behaviour, attitudes, and beliefs*, Lorraine Mazerolle, Adrian Cherney, Elizabeth Eggins, Angela Higginson, Lorelei Hine, Emma Belton, 05 March 2019.

*Multiagency programs with police as a partner for reducing radicalisation to violence*, Lorraine Mazerolle, Adrian Cherney, Elizabeth Eggins, Angela Higginson, Lorelei Hine, Emma Belton, 10 April 2019.

*Effects of opioid‐specific medication assisted therapies on criminal justice and overdose outcomes*, Catherine Strange, Sarah M. Manchak, Cory Haberman, Jordan M. Hyatt, Alisha Desai, 21 May 2019.

## 3 DISABILITY

*Effectiveness of interventions for improving livelihood outcomes for people with disabilities in low‐ and middle‐income countries*, Hannah Kuper, Ashrita Saran, Lena Morgon Banks, Howard White, 12 March 2019.

*Social interventions to improve well‐being of people with mental disorders: global evidence and gap map*, Sherize Merlin Dsouza, Jisha B. Krishnan, Ann Mary Sebastian, Ashrita Saran, 13 March 2019.

*Nutrition status and its relationship with health status in individuals with spinal cord injury*, Jia Li, Devin Drummer, Thomas Hoover, Christian Sidebottom, Rachel Cowan, John‐Paul Tortorich, Cindy Cai, Elizabeth Scalia, Ceren Yarar‐Fisher, 29 March 2019.

*What is the relationship between back shape/posture, balance, falling, and fear of falling in older adults with hyperkyphosis?*, Roongtip Duangkaew, Josette Bettany‐Saltikov, Paul van‐Schaik, Gok Kandasamy, Julie Hogg, 20 March 2019.

*Exercise interventions to improve back shape/posture, balance, falls and fear of falling in older adults with hyperkyphosis*, Roongtip Duangkaew, Josette Bettany‐Saltikov, Paul van‐Schaik, Gok Kandasamy, Julie Hogg, 20 March 2019.

## 4 EDUCATION

*The effectiveness of homework in primary school*, Jennifer Hanratty, Sarah Miller, Aoibheann Brennan‐Wilson, Maria Cockerill, Jenny Davison, Jennifer Roberts, Karen Winter, 13 February 2019.

*School‐based reading interventions for improving reading skills and educational outcomes on primary school students in low‐ and middle‐income countries*, Qiufeng Gao, Huan Wang, Scott Rozelle, Yaojiang Shi, Jason Li, Lifang Zhang, Wei Nie, 11 March 2019.

## 5 INTERNATIONAL DEVELOPMENT

*Effectiveness of transport sector interventions in low‐ and middle‐income countries: an evidence and gap map*, Denny John, Howard White, Nina Blöndal, 26 February 2019.

## 6 KNOWLEDGE TRANSLATION AND IMPLEMENTATION

*Systematic review of methods of reducing risk of bias in the evaluation of knowledge translation strategies for evidence‐informed health policymaking*, Ayat Ahmadi, BaharehYazdizadeh, Leila Doshmangir, Reza Majdzadeh, ShabnamAsghari, 25 February 2019.

## 7 METHODS

*The methodological and reporting characteristics of economic methods and outcomes in Campbell reviews*, Denny John, Pauline Sobiesuo, Devarshi Bhattacharyya, Luke Vale, 05 April 2019.

## 8 SOCIAL WELFARE

*Effectiveness of adult day care centres for improving quality of life in older adults in low‐ and middle‐income countries*, Saritha Susan Vargese, Nisha Kurian, Elsheba Mathew, Daies Idiculla, Geethu Mathew, Sunu Alice Cherian, Denny John, 22 January 2019.

*Book reading for promoting physical and mental health in older adults*, Jorien Laermans, Hans Scheers, Philippe Vandekerckhove, Emmy De Buck, 22 January 2019.

*Friendly visiting by a volunteer for reducing loneliness and social isolation, and improving wellbeing in older adults*, Jorien Laermans, Hans Scheers, Philippe Vandekerckhove, Emmy De Buck, 22 January 2019.

*Interventions for reducing violence against children in low‐ and middle‐income countries: an evidence and gap map*, Prachi Pundir, Ashrita Saran, Howard White, Jill Adona, Ramya Subrahmanian, 04 February 2019.

*Institutional responses to child maltreatment: an evidence and gap map*, Bianca Albers, Caroline Fiennes, Aron Shlonsky, Ludvig Bjørndal, James Hennessy, Joachim Krapels, Robyn Mildon, 14 February 2019.

*Improving access to health and social services for individuals experiencing, or at risk of experiencing, homelessness*, Sarah Miller, Ciara Keenan, Jennifer Hanratty, 21 February 2019.

*Discharge programmes for individuals experiencing, or at risk of experiencing, homelessness*, Jennifer Hanratty, Sarah Miller, Ciara Keenan, 21 February 2019.

*Accommodation‐based interventions for individuals experiencing, or at risk of experiencing, homelessness: a network meta‐analysis*, Ciara Keenan, Sarah Miller, Jennifer Hanratty, 21 February 2019.

*The effectiveness of supported employment for young adults with severe mental illness in improving work outcomes*, Yue Bai, Zhaowen Cheng, Wei Bian, Zhenggang Bai, Jia Li, Iris Chi, 28 February 2019.

*Interventions for adults exposed to war and armed conflict: an evidence and gap map*, Anne Farina, Brandy R. Maynard, 14 March 2019.

*The effectiveness of social protection interventions in low‐ and middle‐income countries: an evidence and gap map*, Latha T, Zinnia Sharma, Linu A John, Ashrita Saran, 19 March 2019.

*Examining the best time of day for exercise*, Meixuan Li, Xiaoqin Wang, Liang Yao, Huijuan Li, Liujiao Cao, Peijing Yan, Xiuxia Li, Kehu Yang, 03 May 2019.

*Effectiveness of road safety interventions: an evidence and gap map*, Dinesh Mohan, Geetam Tiwari, Mathew Varghese, Kavi Bhalla, Denny John, Ashrita Saran, Howard White, 09 May 2019.

